# Cerebrospinal Fluid Hypocretin-1 (Orexin-A) Level Fluctuates with Season and Correlates with Day Length

**DOI:** 10.1371/journal.pone.0151288

**Published:** 2016-03-23

**Authors:** Kim Boddum, Mathias Hvidtfelt Hansen, Poul Jørgen Jennum, Birgitte Rahbek Kornum

**Affiliations:** 1 Molecular Sleep Laboratory, Department of Clinical Biochemistry, Rigshospitalet, Glostrup, Denmark; 2 Danish Center for Sleep Medicine, Department of Clinical Neurophysiology, Rigshospitalet, Glostrup, Denmark; The University of Texas at Austin, UNITED STATES

## Abstract

The hypocretin/orexin neuropeptides (hcrt) are key players in the control of sleep and wakefulness evidenced by the fact that lack of hcrt leads to the sleep disorder Narcolepsy Type 1. Sleep disturbances are common in mood disorders, and hcrt has been suggested to be poorly regulated in depressed subjects. To study seasonal variation in hcrt levels, we obtained data on hcrt-1 levels in the cerebrospinal fluid (CSF) from 227 human individuals evaluated for central hypersomnias at a Danish sleep center. The samples were taken over a 4 year timespan, and obtained in the morning hours, thus avoiding impact of the diurnal hcrt variation. Hcrt-1 concentration was determined in a standardized radioimmunoassay. Using biometric data and sleep parameters, a multivariate regression analysis was performed. We found that the average monthly CSF hcrt-1 levels varied significantly across the seasons following a sine wave with its peak in the summer (June—July). The amplitude was 19.9 pg hcrt/mL [12.8–26.9] corresponding to a 10.6% increase in midsummer compared to winter. Factors found to significantly predict the hcrt-1 values were day length, presence of snow, and proximity to the Christmas holiday season. The hcrt-1 values from January were much higher than predicted from the model, suggestive of additional factors influencing the CSF hcrt-1 levels such as social interaction. This study provides evidence that human CSF hcrt-1 levels vary with season, correlating with day length. This finding could have implications for the understanding of winter tiredness, fatigue, and seasonal affective disorder. This is the first time a seasonal variation of hcrt-1 levels has been shown, demonstrating that the hcrt system is, like other neurotransmitter systems, subjected to long term modulation.

## Introduction

The hypocretin (hcrt, also known as orexin) neuropeptides regulate several homeostatic functions including the sleep/wake cycle, food intake, energy homeostasis, and arousal [1,2]. The hcrt precursor protein is encoded by a single gene from which the two active neuropeptides, hcrt-1 and hcrt-2, are processed. Hcrt neuropeptides are produced exclusively in a small group of neurons, the hcrt neurons, in the lateral hypothalamus [3]. These hcrt neurons project to and activate most of the central nervous system [4], while integrating signals about metabolic and nutritional status, emotional and motivational state, and expectance of reward to evoke the appropriate level of arousal [5]. Dysregulation of hcrt levels has severe consequences for the organism: loss of hcrt neurons causes the sleep disorder, narcolepsy, which is characterized by excessive daytime sleepiness and decreased sleep quality [6]. Patients with this disorder also experience metabolic disturbances and autonomic dysfunction [7]. Narcolepsy with loss of hypocretin, also called Narcolepsy Type 1 can be diagnosed by measuring hcrt-1 levels in cerebrospinal fluid (CSF) [8]. In Narcolepsy Type 1 hcrt-1 is absent in the CSF or concentrations are very low.

Hcrt-1 peptide level in the brain is under complex regulation, and has interestingly been shown to respond to playful activities and social interaction [9–11]. Furthermore, recent animal studies indicate that the hcrt system is also under the influence of light [12,13]. In Northern countries (as well as countries in the far South) the day length varies substantially over the year. In Denmark it ranges from 7 to 18 hours. The dark winters are associated with tiredness, fatigue, reduced sleep quality, and decreased mood [14–16].These findings led us to speculate whether the hcrt system is affected by seasons and if hcrt activity in humans is reduced during dark winters. We, therefore, investigated hcrt-1 levels in CSF of clinical samples collected from patients at the Danish Center for Sleep Medicine over a period of almost 4 years.

Hcrt-1 levels can only be reliably detected in the CSF, most often obtained by lumbar puncture. In contrast to many other national strategies, lumbar puncture is a mandatory part of the clinical evaluation of hypersomnia in the Danish healthcare system. We were, therefore, able to collect a dataset stemming from 227 individuals with normal hcrt-1 levels (measured over a period of 45 months) and including their biometric and clinical data. Further, we have, in collaboration with the National Danish Institute of Metrology, obtained data on the local climate conditions matching the time points of individual CSF collections. From this dataset we examined seasonal levels of CSF hcrt-1 and possible correlating factors.

## Material and Methods

A more detailed description of study measures, design, and analysis is provided in the supplementary materials in S1 File. The final dataset can be found in S2 File.

### Subjects

Approval of the study was granted by the Danish board of Health and the Danish Data Protection Agency (#3-3013-898/1). All human data included in this study was obtained from patients’ medical records including CSF hcrt-1 levels. CSF had been collected and analysed as a part of the clinical evaluation of hypersomnia at the Danish Center for Sleep Medicine (DCSM), Department of Clinical Neurophysiology, Rigshospitalet, Glostrup, Denmark. By approval, no informed consent was given by the participants, as data were obtained from a clinical registry and analysed anonymously.

### Hcrt measurements

CSF was collected between 09:00 and 12:00 AM, a time window in which other studies have found no significant change in the hcrt-1 level when measured in the amygdala [11] or less than 5% change from a 24 hour in CSF drawn continuously with an intrathecal catheter [17]. The CSF was cooled on ice and stored within 30 min at—80°C. Hcrt-1 levels were analysed by the standard radioimmunoassay (RIA, Phoenix Pharmaceuticals, CA, USA). Assay quality was monitored by the internal positive control sample included in the assay. Intra-assay variability was accessed by including a Hcrt-1 control from the previous assay in each assay, and additionally, an external reference sample of pooled CSF from healthy individuals was included for normalisation of values between assays and adjustment to the clinical standard level of CSF hcrt-1 [18]. The external reference CSF sample was originally donated by Dr. E. Mignot, Stanford Center for Sleep Sciences and Medicine, Stanford University, USA.

### Clinical data

Patients who had undergone a lumbar puncture at DCSM in the period January 2011 –September 2014 were considered for the present study (n = 382). Exclusion criteria were a diagnose of “narcolepsy with low hypocretin”, or hypocretin level <110 pg/mL (corresponding to”Narcolepsy Type 1” according to the International Classification of Sleep Disorders, Third addition [19]), intermediate hypocretin 110–220 pg/mL or no data (i.e. premature termination or absence of patient from clinical examination). Most patients were form the greater Copenhagen area.

### Climate data

Climate data for each day in the study period (day length, average temperature, snow coverage, snow depth, and hours of sunshine) were retrieved from National Danish Institute of Metrology (DMI) upon request. The data were from the Copenhagen area (55°40′N 12°34′E). Since Denmark is only 452x368 km in size (including a remote island in the Baltic sea), weather conditions are generally similar across the entire country.

### Data analysis

To study seasonal variation, data on CSF Hcrt-1 levels were grouped according to sampling month. Grouped data were fitted with a sine-wave function (wavelength = 12 months) with a nonlinear, least-squares fitting method (Prism 5, GraphPad, CA, USA).

In order to access relationship between hcrt-1 levels and relevant variables, we performed a multiple regression analysis (IBM SPSS Statistics 19, IBM Corp., Armonk, NY). Variables considered for the analyses were: Hcrt-1 level, BMI, gender, age, diagnose, leukocyte count, CRP (C-reactive protein) level, MSLT/PSG variables and several climate factors. Because the climate data where highly correlated (S1 Table), these factors could not be included together in the multiple regression analysis. Instead we analysed them in separate models and compared the models. Separate models included the following climate variables: Day length (hours pr. actual day and average hours the preceding three weeks), slope of day length change (min/day), sunshine hours (hours the day before CSF sampling and average hours the preceding three weeks), temperature (average °C across 24 h on the day of CSF sampling and average temperature the preceding three weeks), and snow (yes/no). Days with at least 50% snow coverage or at least 25% snow coverage plus snow the preceding two days were categorized as days with snow, while the rest where called days with no snow (snow n = 22, no snow n = 205). We accounted for diagnose by including a factorial variable with 4 categories: Narcolepsy Type 2, idiopathic hypersomnia, sleep apnea, other.

In each model assumptions of linearity, independence of errors, homoscedasticity, unusual points, and normality of residuals were tested. One outlier was removed from the analysis. This was a patient with very high levels of hcrt-1 levels, who had a diagnosis of Narcolepsy Type 2 and major depression.

Leukocyte count, CRP (C-reactive protein) levels, MSLT, and PSG data were only available in a subset of patients, so these variables were tested in separate models including the cofactors mentioned above.

## Results

### CSF hcrt-1 levels fluctuate with season

**Fig 1:**
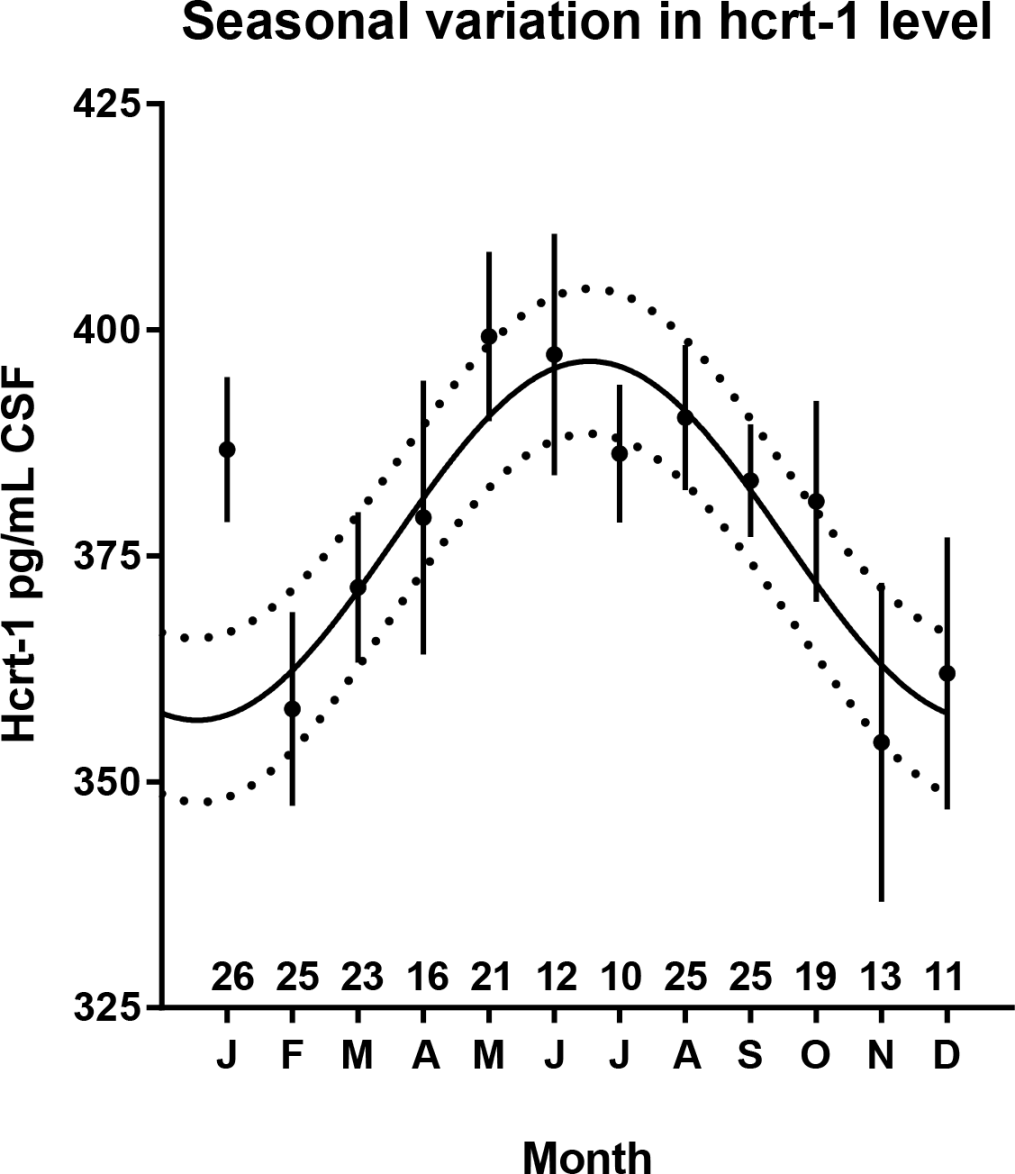
Seasonal variation in CSF hcrt-1 levels in human individuals. Sinusoidal fitted curve to monthly average of patient CSF hypocretin-1 levels, excluding January. Dashed lines represent 95% confidence interval. The inserted numbers above x-axis equals the number of data points for each month.

A summary of the data extracted from patient hard copy journals and national electronic health records can be seen in Table 1. The table also lists the climate factors included in the study. In total 227 individuals were included in the study. The monthly average of CSF hcrt-1 values was found to vary between months, revealing a seasonal variation in the hcrt-1 levels (Fig 1) with a peak in the Danish summer months and a minimum at winter. A 12 month sine wave predicted the data (*r*^2^ = 0.71) with an amplitude of 19.9 pg/mL [12.8; 26.9] when data from January were excluded. This oscillation corresponds to a 10.6% change which is close to the magnitude of diurnal variation found in healthy subjects (16). The month with the lowest average was November (354.4 pg/ml, n = 13), the highest average was found in May (399.3 pg/ml, n = 21), corresponding to an increase of 12.7%. However, January data did not follow this trend with the average hcrt-1 level in January being higher than expected from the model (Fig 1).

**Table 1:** Summary of variables in the dataset.

	N	Mean	Min	Max	SEM
**sex (M/F)**	96/131				
**Diagnose (NC2/IH/SA/other)^a^**	41/103/17/66				
**Age (years)**	227	39.9	15	78	15.5
**BMI**	227	24.9	16.4	43.8	4.67
**Hcrt-1 (pg/mL)**	227	380.4	232	536	47.5
**Sun (h/day)**	227	4.2	0	15.5	4.21
**Day length (min/day)**	227	723	422	1052	199
**Temperature (°C /day)**	227	8.79	-4.8	23.2	6.74
**Snow (yes/no)**	22/205				
**Leukocyte (x10^9^/L)**	182	6.59	3.2	12.8	1.94
**CRP (mg/L)**	112	5.91	<5^b^	26	3.66
**MSLT SOREMs (0/1/2/3/4/5)**	118/36/34/19/6/3				
**MSLT sleep latency (min)**	216	9.14	0.8	30	5.76
**PSG Total sleep (min)**	217	427	98	1012	103

a) NC2: Narcolepsy Type 2, IH: idiopathic hypersomnia, SA: sleep apnea.

b) Reference interval limit was 5.

### CSF hcrt-1 levels correlate with day length

To elucidate possible factors causing the variation in CSF hcrt-1 levels, we included the following variables in our analysis: BMI, gender, age, diagnosis, leukocyte count, C-reactive protein (CRP) level, multiple sleep latency test (MSLT) sleep onset REM periods (SOREMs), MSLT sleep latency, polysomnography (PSG) total sleep length, day length, slope of day length change, sunshine hours (direct radiation exceeding 120 W/m^2^), and presence of snow. As the hcrt system might by dysregulated in Narcolepy Type 2, despite apparent normal hcrt-1 levels, we decided to include a variable accounting for this diagnosis. Age, gender and diagnosis had no effect on hcrt-1 levels in any of the models, while BMI had a borderline significant effect with lower BMI levels predicting higher hcrt-1 (Table 2).

**Table 2:** Predictors of CSF hcrt-1 level. Summary of Multiple Regression Analysis.

Variable	B	SE_B_	β	*p*-value
**Intercept**	423.607	32.805		
**Age**	.034	.211	.011	.873
**Gender**	4.319	6.406	.046	.501
**BMI**	-.973	.680	-.097	.154
**Diagnosis^a^**	.135	2.687	.003	.960
**Day length /3 weeks**	.216	.048	.327	.000011
**Snow (Y/N)**	30.350	11.048	.194	.007
**Days after Christmas^b^**	-2.762	1.033	-.186	.008

a) Narcolepsy Type 2, idiopathic hypersomnia, sleep apnea, or other diagnosis;

b) Days longer than 30 days from Christmas Day were given the value 30.

B = unstandardized regression coefficient; SE_B_ = Standard error of the coefficient; β = standardized coefficient. N = 222.

**Fig 2:**
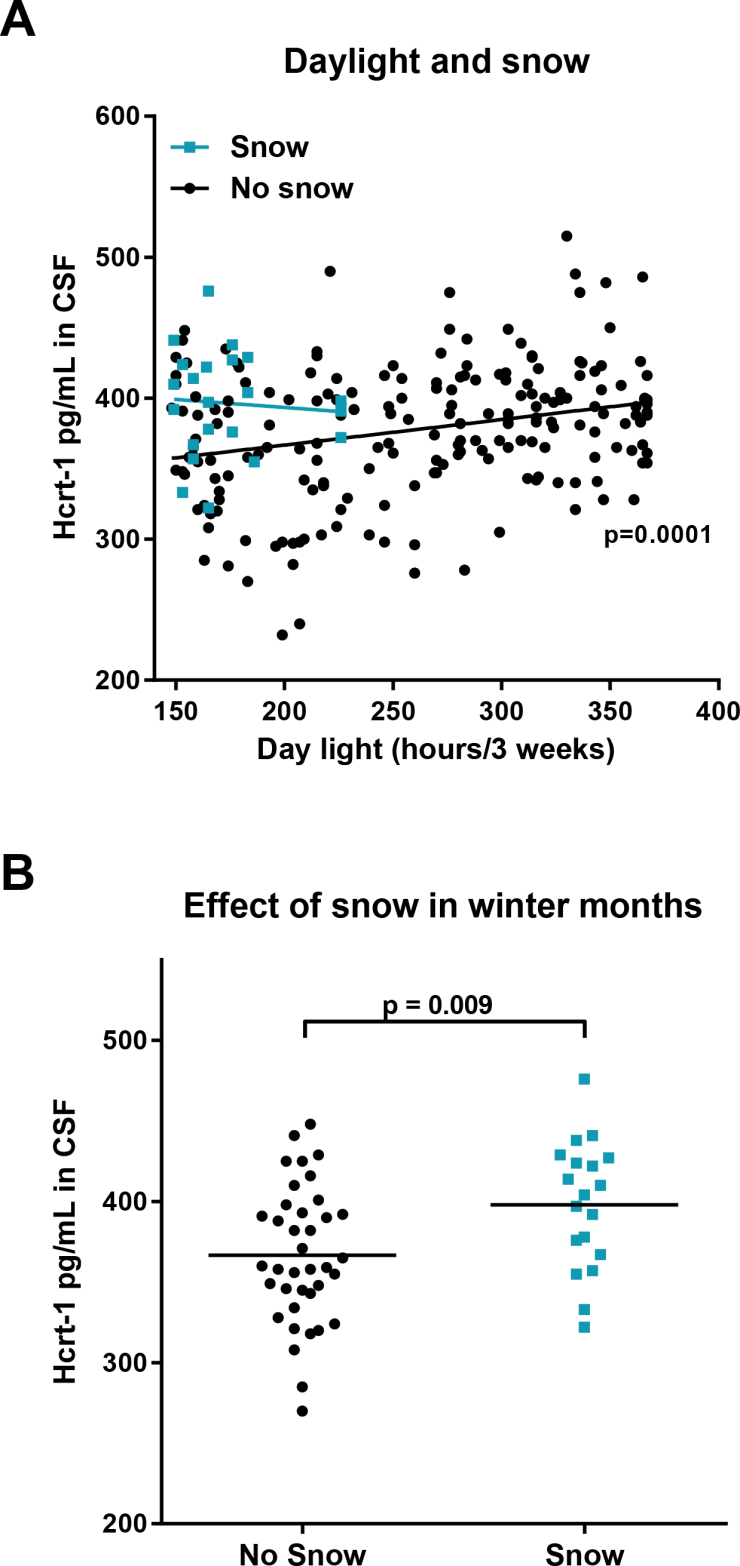
CSF hcrt-1 correlates with day light levels and presence of snow. A) The relationship between CSF hcrt-1 levels and average day length the preceding three weeks, divided in groups dependant on the presence of snow. In the samples taken on days without snow, there was a significant correlation (linear regression p = 0.0001) between day light and the hcrt-1 level, which was not found in the samples taken on days with snow. B) The effect of snow on hcrt-1 levels in winter month (Dec-Feb). The average hcrt-1 level is significant higher (student t-test, p = 0.009) when CSF sampling was performed on days with snow than on days without snow.

Our analysis revealed significant effects of day length, sunshine, and temperature, and showed that the model including average day length the preceding three weeks best predicted CSF hcrt-1 values (S2 Table). We included both the values for the day before CSF sampling and also average values for the preceding 3 weeks in order to take a possible longer lasting, modulating influence into account. The final model (including age, gender, BMI, diagnosis, average day length of the preceding three weeks, presence of snow, and number of days from Christmas) statistically significantly predicted CSF hcrt-1 values, *F*(7,214) = 4.106, *p* = 0.0003, R^2^ = 0.118. In this model longer day length, presence of snow, and proximity to Christmas all significantly predicted higher levels of hcrt-1 in CSF (*p* = 11x10^-6^, *p* = 0.007, and *p* = 0.008 respectively, Table 2 and Fig 2A). None of the clinical MSLT or PSG parameters correlated significantly with hcrt-1 concentrations (S3 Table). There were also no predictive values of leukocyte count and CRP level in the subset of patients where this information was available (S4 and S5 Tables).

### Higher hcrt-1 levels than expected in January

**Fig 3:**
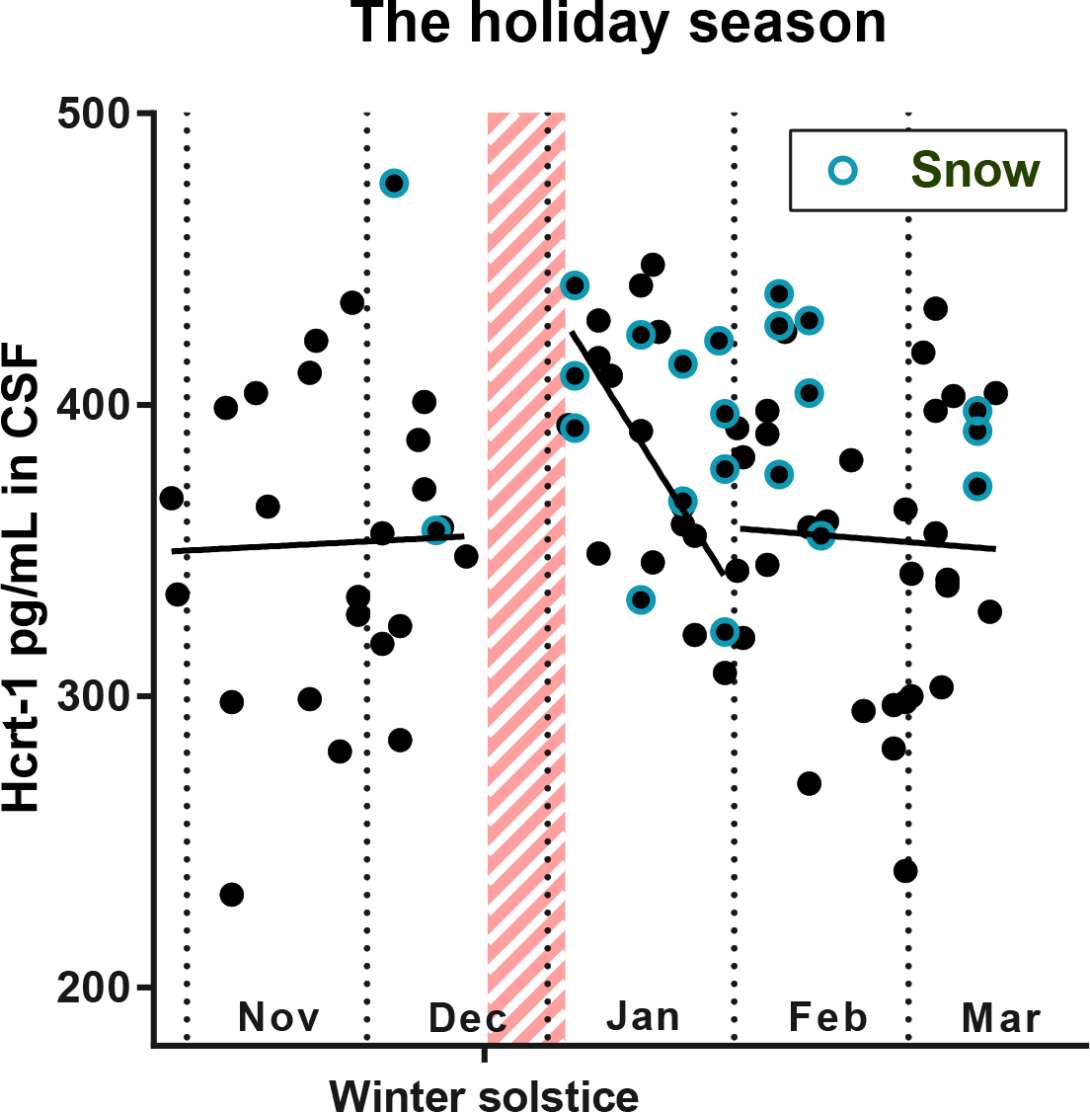
CSF hcrt-1 levels around Christmas vacation in Denmark. Shown are linear regression fits of data excluding days of snow and divided into three parts: Nov-Dec (non-significant), Jan (p = 0.03), and Feb-March (non-significant). The Christmas vacation is marked with red and data sampled on days with snow are marked with a blue circle.

When comparing the average hcrt-1 level, measured on days with snow in the period December to February, the level was significantly higher than the level measured on days without snow coverage in the same period (*p* = 0.006; Fig 2B). This effect was present in all winter months, except beginning of January, where levels of hcrt-1 were higher than expected from the model also on days with no snow and very little sunlight (Fig 3). The level of hcrt-1 did, however, correlate significantly with the number of days between the CSF sample was taken and the Christmas holidays (Table 2 and Fig 3).

## Discussion

Here we provide data showing a seasonal variation in hcrt-1 peptide level in human CSF. To our knowledge, this is the first time it has been shown that hcrt-1 levels exhibit a seasonal rhythm. We found a 10–12% change in the mean hcrt-1 level from winter to summer, a value which is of the same magnitude as seen in the diurnal variation of CSF hcrt-1 in humans [17,20], and thus expected to have functional significance. We furthermore report correlations between hcrt-1 levels and day length. Data from January did not follow the general trend in the data, an effect partly explained by the presence of snow.

All the samples were taken within a clearly defined timespan of the day, where other studies have shown that the hcrt level varies only minimally [17] or insignificant [11] to minimize the impact of the diurnal variation of hcrt-1. Our data show an overall seasonal change with large individual variation suggesting that besides daily fluctuations, the hcrt system is subject to a more long term modulation of baseline signalling, as is the case for other neurotransmitter systems such as the serotonin system [21,22]. This is also supported by studies showing experience-dependent synaptic plasticity and remodeling of hcrt neurons in mice [23,24].

Curiously, we did not find any correlations between hcrt-1 levels in CSF and the included measures of sleep length and daytime sleepiness. It is known from existing literature and general clinical experience that low hcrt-1 in CSF predicts a short MSLT sleep latency and several SOREMs. What is clear from our data is that for patients with a CSF hcrt-1 level >220 pg/ml such correlation is no longer present. There could be several explanations for this observation. It might be that the MSLT is not sensitive enough to pick up subtle changes in daytime sleepiness resulting from having a hcrt-1 level in the low end of the normal range. It is also possible that severe daytime sleepiness and REM disturbances do not occur at all before hcrt-1 levels become abnormally low.

### Climate factors correlate with hcrt-1 levels

Copenhagen is located around 56° northern latitude which gives rise to large variations in day length with about 7 hours in the winter and up to 18 hours during summer. Taking the weather (cloud occurrence) into account, the actual hours of daily sunshine can vary even further. Our data show that both day length and hours of sunshine predict CSF hcrt-1 levels, and the model including day length predicted the hcrt-1 levels slightly better than the model including sunshine. Interestingly, average day length data and average sunlight levels over three weeks predicted CSF hcrt-1 levels better than data from the day before testing only, suggesting that the lumbar CSF hcrt-1 values do not reflect fast fluctuations of central hcrt activity in response to day to day weather conditions, but rather reflecting a long term average over several weeks. This is supported by a previous study showing that hcrt-1 measurements from the same individual taken 1–2 weeks apart were highly correlated [20]. On top of this baseline 5–10% diurnal fluctuation is however still seen [17,20], so a shorter acting clearance of hcrt-1 from the CSF is also present. This has also been demonstrated in dogs, where an increase in CSF hcrt-1 following sleep deprivation was not sustained beyond 24 hours [10].

### Hcrt-1 levels and light

A possible explanation for our finding of seasonal changes in hcrt-1 levels could be that daylight levels influence hcrt-1 signalling. The independent effect of snow we observe in our dataset could also be explained as a consequence of increased light exposure as a result of reflection from a snow covered surface. This lighting effect would be expected to be give largest impact in periods with very little daylight, and this is also what we see in the dataset. As can be seen in Fig 2, the difference in hcrt-1 levels between days with snow and days without is greater in periods with very little sun.

An influence of light on hcrt neurons has already been suggested by animal studies. It has been shown that in the diurnal species, the grass rats, a light pulse stimulates immediate-early gene activity in hcrt neurons [25], whereas dim light housing conditions lowers hcrt immunoreactivity in the brain [13]. In the nocturnal mouse, in contrast, dark pulses (arousal cues for nocturnal species), can activate hcrt neurons [26]. Despite hcrt neurons being primarily active in the dark phase in mice, it has also been shown that light activates mouse hcrt neurons during positively reinforced tasks [12]. This finding is consistent with the lack of the arousing effect of light in humans suffering from narcolepsy [27]. Since no direct pathway from the retina to the hcrt neurons has been found, this is likely an indirect effect.

### Hcrt-1 levels and arousal

Another possible explanation for our finding is that the overall activity and arousal level during the day is higher in the summer months and on days with snow causing an increased release of hcrt-1 and thus higher CSF levels. The hcrt-producing neurons link forebrain structures involved in the processing of emotion and motivation, such as the amygdala, with brainstem regions, such as locus coeruleus, which regulate wakefulness and reward [5], and cues associated with rewards and arousal stimulate the hcrt neurons [28,29]. For example, it has been shown that yard play produces a substantial increase in CSF Hcrt-1 level in normal dogs, whereas comparable treadmill locomotion did not increase Hcrt-1 level beyond baseline [9]. It is therefore possible that increased purposeful behaviour with longer days is the underlying mechanism behind our finding.

Many studies have indeed concluded that physical activity levels change with seasonal changes in weather conditions [30]. The patterns are heavily dependent on local weather conditions thus local data are crucial: A activity study of 730 Danish children, using actigraphy, found that daytime activity was higher in spring and sleep length was shorter compared to both autumn and winter [31]. This is supported by a study from Norway reporting the same pattern, with higher activity levels in spring [32]. However, if the amount of physical activity was the primary driver of the observed seasonal variation, we would expect higher hcrt-1 levels in spring compared to fall independent of day length. This is not the case in our dataset (Fig 1, compare March-April to September-October where day lengths are similar). Since we do not have any measures of activity in our cohort, we cannot make any final conclusions regarding this hypothesis.

### Hcrt-1 and social interaction

Increased levels of hcrt-1 in humans have been found during social interactions and in connection with social-induced positive emotions [11]. Indeed, hcrt neurons receive abundant input from the limbic system [33] and several papers have provided experimental evidence for an involvement of the hcrt system in emotion and emotional memory [28,34,35]. It is often speculated that that activation of hcrt neurons by the limbic system maintains wakefulness during emotional arousal. It is therefore possible that seasonal changes in social interaction patterns also account for some of the variation seen in our dataset, most noteworthy the increased hcrt-1 levels in January following the Christmas vacation. Social interaction between a patient and relatives has already been shown to increase hcrt activity [11], thus it could be interesting to study whether larger social gatherings also increase hcrt activity and if so, to what extent.

### Possible consequences of seasonal fluctuation in hcrt-1 levels

It is well established that hcrt neurons can excite serotonin neurons [5,36]. Serotonin-related conditions such as depression show a clear seasonal pattern in humans, which is consistent with plasma serotonin (5-HT) levels undergoing marked changes throughout the year, with maximum values during the summer [21]. Dampening of the natural diurnal hcrt-1 variation in CSF has been found in depressed subjects [17], and people who have attempted suicide have reduced levels of Hcrt-1 in their CSF [37], suggesting that a poorly functioning hcrt system could play a role in mood disorders. It is however still controversial whether hcrt-1 levels are lower in depressed patients, as stress induced depression is sometimes linked to an increase in hcrt activity [38].

Decreased day length and dark winters increase the risk of seasonal affective disorder (SAD) [15,39,40]. It has previously been shown that SAD patients have lower physical activity levels and a blunted 24-h activity rhythm compared to healthy controls. These abnormalities were completely reversed by bright day light therapy [41]. Given that animal data shows an influence of light on hcrt signalling, it is possible that the seasonal changes in hcrt-1 levels at northern latitudes play a role in the ethiology of SAD.

### Limitations

A limitation to this study is that subjects included in this study are not from the normal healthy population. For ethical and practical reasons, the CSF hcrt-1 levels were obtained as a part of a clinical evaluation of hypersomnia. All individuals included must therefore have experienced some sort of subjective sleep disturbance. However, neither diagnosis nor any of the clinical parameters included correlated significantly with hcrt-1 levels, indicating that our finding represents a general trend.

## Conclusion

In conclusion, this study demonstrates a clear seasonal variation in human CSF hcrt-1 levels, with a peak in the summer months and lowest levels during winter. This shows that the activity of the hcrt system changes over the year. We found a significant correlation with day length and presence of snow. Interestingly, an unknown factor caused hcrt-1 levels to be higher in January than expected from the model. Further, cumulative day length over three weeks better predicted hcrt-1 levels compared to day length on the day of CSF sampling. This is suggestive of the presence of long term modulating effects on baseline activity in the hcrt system.

These findings add valuable knowledge to our understanding of the role and regulation of the hcrt system, and they have several possible interpretations. Seasonal changes in leisure activities, light exposure, and social interaction could all possibly influence the activity of the hcrt system. Our findings have implications for understanding the mechanisms of seasonal changes in arousal and mood, and could point towards the hcrt system as an important mediator or modifier of day light effects on other neurotransmitter systems such as the monoaminergic systems. Seasonal variations in hcrt-1 levels should further be taken into account when interpreting CSF hcrt-1 values in clinical practice.

## Supporting Information

S1 File Supplementary methods. (PDF)

S2 File Full dataset. (XLSX)

S1 Table Correlations among climate factors. (DOCX)

S2 Table Summary of Multiple Regression Models including different climate factors. (DOCX)

S3 Table No predictive value of MSLT and PSG data. Summary of Multiple Regression Analysis.
(DOCX)

S4 Table No predictive value of leucocyte count. Summary of Multiple Regression Analysis.
(DOCX)

S5 Table No predictive value of C-reactive protein level. Summary of Multiple Regression Analysis.
(DOCX)
