# Supplementary material for: Cerebrospinal Fluid Hypocretin-1 (Orexin-A) Level Fluctuates with Season and Correlates with Day Length
Source: PLoS One. 2016 Mar 23;11(3):e0151288. doi: 10.1371/journal.pone.0151288 (PMC4805193; doi:10.1371/journal.pone.0151288)
Supplement: S1 File — (PDF) [file pone.0151288.s001.pdf]

Supplementary Methods

Subjects

Approval of the study was granted by the Danish board of Health and the Danish Data Protection Agency (#3-3013-898/1). CSF was collected from patients with possible hypersomnias as a part of the clinical evaluation of hypersomnia at the Danish Center for Sleep Medicine (DCSM), Department of Clinical Neurophysiology, Rigshospitalet, Glostrup, Denmark.

Hcrt measurements

CSF was collected between 08:00 and 12:00 AM, cooled on ice and stored within 30 min at - 80° C. Hcrt-1 was analysed in 100 µL crude CSF by the standard radioimmunoassay (RIA, Phoenix Pharmaceuticals, CA, USA). The samples were thawed on ice and briefly vortexed before added to the RIA. Samples were measured blindly as duplicates and result means were calculated. The standard curve range was 10-1280 pg/mL, and the detection limit was 20 pg/mL. Assay quality was monitored by the internal positive control sample included in the assay kit according to manufactures guideline and if the value was >5% the samples were reanalysed. Intra-assay variability was accessed by including a Hcrt-1 control from the previous assay in each assay to estimate the day-to-day variability which did not exceeded 9%. Additionally, an external reference sample of pooled CSF from healthy individuals was included for normalisation of values between assays. The external reference CSF sample, included in every assay, was originally donated by Dr. E. Mignot, Stanford Center for Sleep Sciences and Medicine, Stanford University, USA. Using this reference all measurements were normalized between assays and adjusted to the clinical standard level of CSF hcrt-1 as defined at Stanford University. No drift in average hcrt-1 values was observed over the 4 years.

Clinical data

The personal identification number, individual to every citizen in Denmark, enables matching of every patient to the Danish Health Registers, and by taking advantage of this we obtained matched data on CSF hcrt-1 level and other relevant clinical parameters. Patients who had undergone a lumbar puncture at DCSM in the period January 2011 – September 2014 were considered for the present study (n=382). The medical history of the individual patient were obtained in the form of hard copy journals or extracted from the national electronic health records. Exclusion criteria were a diagnose of "narcolepsy with low hypocretin", or hypocretin level <110 pg/mL (corresponding to "narcolepsy type 1" according to the International Classification of Sleep Disorders, Third addition). Patients with intermediate hypocretin 110-220 pg/mL were also excluded since these likely do not have a normal regulation of hypocretin. Finally patients with no data (e.i. premature termination or absence of patient from clinical examination) were excluded.

227 samples were included in the study and by January 2015 the patients had the following diagnosis: Narcolepsy type 2 (41), Idiopathic hypersomnia (103), Secondary narcolepsy (4), secondary hypersomnia (5), Sleep apnea (17), REM sleep behaviour disorder (10), restless legs syndrome (3), circadian sleep disturbance (3), Kleine Levin Syndrome (2), epilepsy (2), multiple sclerosis (1), NREM parasomnia (1), unspecific symptoms/inconclusive (35).

The clinical data extracted from the records were: Sex, age, diagnose, hcrt-1 level, body mass index (BMI), Leukocyte count, C reactive protein (CRP), Multiple sleep latency test (MSLT) sleep onset REM periods (SOREMs), MSLT sleep latency, polysomnography (PSG) total sleep length, and date of CSF sampling.

Climate data

Climate data for each day in the study period (day length, average temperature, snow coverage, snow depth, and hours of sunshine) were retrieved from National Danish Institute of Metrology (DMI) upon request. The data were from the Copenhagen area (55°40'N 12°34'E), in which the Danish Center for Sleep Medicine is located.

From the climate data we had access to the following variables: day length as minutes between sun rise and sun set; average temperature over 24 hours in degrees Celsius; hours of sunshine (direct radiation exceeding 120 W/m^2) between sun rise and sun set with 1 decimal; a categorical estimation of the snow coverage of the ground (0-4, with 4 representing full coverage); snow depth in centimetres. Snow is a rare event in Denmark so only 26 data points were obtained on days with snow out of a total of 227. Snow was included in the analysis because of the possibility of stronger daylight levels under the influence of snow, we therefore decided to include snow in the analysis as a binary factor. Days with at least 50% snow coverage or at least 25% snow coverage plus snow the preceding two days were categorized as days with snow, while the rest where called days with no snow (snow n=22, no snow n=205). Regarding day length and sunshine, we included both the values for the day before CSF sampling and also average values for the preceding 3 weeks to take into account possible longer lasting modulating influence. Finally the slope of the change in day length was calculated.

Data analysis

To study seasonal variation, data on CSF Hcrt-1 levels were grouped according to sampling month. Grouped data was fitted with a sine-wave function (wavelength = 12 months):

$$Y = Amplitude * \sin((2 * \pi / Wavelength) * x + PhaseShift) + mean$$

with a nonlinear, least-squares fitting method (Prism 5, GraphPad, CA, USA).

In order to access relationship between hcrt-1 levels and relevant variables, we performed a multiple regression analysis (IBM SPSS Statistics 19, IBM Corp., Armonk, NY). Variables considered for the analyses were: Hrct-1 level, BMI, gender, age, diagnose, leukocyte count, CRP (C-reactive protein) level, MSLT/PSG variables and several climate factors. As the hypocretin system might by dysregulated in Narcolepy Type 2, despite apparent normal hcrt-1 levels, we decided to include a variable accounting for this diagnose. This was a factorial variable with 4 categories: Narcolepsy Type 2, idiopathic hypersomnia, sleep apnea, other. Because the climate data where highly correlated (Table S1), these factors could not be included together in the multiple regression analysis. Instead we analysed them in separate models and compared the models. In each model assumptions of linearity, independence of errors, homoscedasticity, unusual points, and normality of residuals were tested. One outlier was removed from the analysis. This was a patient with very high levels of hcrt-1 levels, who had a diagnosis of narcolepsy type 2 and major depression.

Leukocyte count, CRP (C-reactive protein) levels, MSLT, and PSG data were only available in a subset of patients, so these variables were tested in separate models including the cofactors mentioned above.
