# Supplementary material for: Cerebrospinal Fluid Hypocretin-1 (Orexin-A) Level Fluctuates with Season and Correlates with Day Length
Source: PLoS One. 2016 Mar 23;11(3):e0151288. doi: 10.1371/journal.pone.0151288 (PMC4805193; doi:10.1371/journal.pone.0151288)
Supplement: S1 Table — (DOCX) [file pone.0151288.s003.docx]

**Table S1**

Correlations among climate factors.

|  | | Temp | Temp (3w) | Sun | Sun (3w) | Day length | Day length (3w) | Slope day | Snow |
| --- | --- | --- | --- | --- | --- | --- | --- | --- | --- |
| Temperature | Pearson | 1 | .892^**^ | .469^**^ | .696^**^ | .751^**^ | .842^**^ | -.542^**^ | -.531^**^ |
|  | Sig. |  | .000 | .000 | .000 | .000 | .000 | .000 | .000 |
| Temp (/3 weeks) | Pearson | .892^**^ | 1 | .358^**^ | .625^**^ | .662^**^ | .792^**^ | -.716^**^ | -.447^**^ |
|  | Sig. | .000 |  | .000 | .000 | .000 | .000 | .000 | .000 |
| Sun (h day before) | Pearson | .469^**^ | .358^**^ | 1 | .591^**^ | .569^**^ | .558^**^ | -.028 | -.140^*^ |
|  | Sig. | .000 | .000 |  | .000 | .000 | .000 | .677 | .035 |
| Sun (/3 weeks) | Pearson | .696^**^ | .625^**^ | .591^**^ | 1 | .874^**^ | .863^**^ | -.070 | -.294^**^ |
|  | Sig. | .000 | .000 | .000 |  | .000 | .000 | .295 | .000 |
| Day length (min) | Pearson | .751^**^ | .662^**^ | .569^**^ | .874^**^ | 1 | .978^**^ | -.036 | -.330^**^ |
|  | Sig. | .000 | .000 | .000 | .000 |  | .000 | .589 | .000 |
| Day length (/3 weeks) | Pearson | .842^**^ | .792^**^ | .558^**^ | .863^**^ | .978^**^ | 1 | -.243^**^ | -.369^**^ |
|  | Sig. | .000 | .000 | .000 | .000 | .000 |  | .000 | .000 |
| Slope day light | Pearson | -.542^**^ | -.716^**^ | -.028 | -.070 | -.036 | -.243^**^ | 1 | .236^**^ |
|  | Sig. | .000 | .000 | .677 | .295 | .589 | .000 |  | .000 |
| Snow (Y/N) | Pearson | -.531^**^ | -.447^**^ | -.140^*^ | -.294^**^ | -.330^**^ | -.369^**^ | .236^**^ | 1 |
|  | Sig. | .000 | .000 | .035 | .000 | .000 | .000 | .000 |  |

**. Correlation is significant at the 0.01 level (2-tailed). *. Correlation is significant at the 0.05 level (2-tailed).
