# Supplementary material for: Cerebrospinal Fluid Hypocretin-1 (Orexin-A) Level Fluctuates with Season and Correlates with Day Length
Source: PLoS One. 2016 Mar 23;11(3):e0151288. doi: 10.1371/journal.pone.0151288 (PMC4805193; doi:10.1371/journal.pone.0151288)
Supplement: S2 Table — (DOCX) [file pone.0151288.s004.docx]

**Table S2**

Summary of Multiple Regression Models including different climate factors.

| Model | Climate variable | *F* | *p* (model) | R^2^ | Β (variable) | *p* (variable) |
| --- | --- | --- | --- | --- | --- | --- |
| 1 | Day length (min) | 2.671 | 0.033 | 0.047 | 0.187 | 0.006 |
| 2 | Average day length (/3 weeks) | 2.736 | 0.030 | 0.048 | 0.190 | 0.005 |
| 3 | Sun (h preceeding day) | 0.986 | 0.416 | 0.018 | 0.074 | 0.276 |
| 4 | Sun (/3 weeks) | 2.535 | 0.041 | 0.045 | 0.180 | 0.007 |
| 5 | Temperature (average °C /24h) | 2.072 | 0.086 | 0.037 | 0.157 | 0.020 |
| 6 | Temperature (/3 weeks) | 2.523 | 0.042 | 0.044 | 0.180 | 0.008 |

All models included age, gender, and BMI.
