# Supplementary material for: Cerebrospinal Fluid Hypocretin-1 (Orexin-A) Level Fluctuates with Season and Correlates with Day Length
Source: PLoS One. 2016 Mar 23;11(3):e0151288. doi: 10.1371/journal.pone.0151288 (PMC4805193; doi:10.1371/journal.pone.0151288)
Supplement: S3 Table — Summary of Multiple Regression Analysis. (DOCX) [file pone.0151288.s005.docx]

**Table S3.**

No predictive value of MSLT and PSG data. Summary of Multiple Regression Analysis.

| Variable | B | SE_B_ | β | *p*-value |
| --- | --- | --- | --- | --- |
| Intercept | 447.574 | 41.874 |  |  |
| Age | .093 | .235 | .029 | .695 |
| Gender | 8.489 | 6.858 | .089 | .217 |
| BMI | -.816 | .704 | -.080 | .248 |
| Day length /3 weeks | .202 | .050 | .302 | .000071 |
| Snow | 27.642 | 12.168 | .165 | .024 |
| Days after Christmas | -3.564 | 1.191 | -.214 | .003 |
| MSLT SOREMPs | -4.218 | 2.885 | -.111 | .145 |
| MSLT sleep latency | .684 | .599 | .082 | .255 |
| PSG total sleep | -.014 | .031 | -.031 | .644 |

B = unstandardized regression coefficient; SE_B_ = Standard error of the coefficient; β = standardized coefficient. N=208. *F*(9,198) = 3.645, *p* = 0.0003, R^2^ = 0.142
