# Supplementary material for: Cerebrospinal Fluid Hypocretin-1 (Orexin-A) Level Fluctuates with Season and Correlates with Day Length
Source: PLoS One. 2016 Mar 23;11(3):e0151288. doi: 10.1371/journal.pone.0151288 (PMC4805193; doi:10.1371/journal.pone.0151288)
Supplement: S4 Table — Summary of Multiple Regression Analysis. (DOCX) [file pone.0151288.s006.docx]

**Table S4**

No predictive value of leucocyte count. Summary of Multiple Regression Analysis.

| Variable | B | SE_B_ | β | *p*-value |
| --- | --- | --- | --- | --- |
| Intercept | 474.898 | 47.106 |  |  |
| Age | -.007 | .226 | -.002 | .974 |
| Gender | 9.395 | 7.123 | .098 | .189 |
| BMI | -1.339 | .756 | -.131 | .078 |
| Day length /3 weeks | .271 | .053 | .401 | .000001 |
| Snow | 45.174 | 13.171 | .256 | .001 |
| Days after Christmas | -4.905 | 1.599 | -.230 | .003 |
| Leukocyte count | .496 | 1.760 | .020 | .778 |

B = unstandardized regression coefficient; SE_B_ = Standard error of the coefficient; β = standardized coefficient. N=178. *F*(7,170) = 5.240, *p* = 0.00002, R^2^ = 0.177.
