# Supplementary material for: Cerebrospinal Fluid Hypocretin-1 (Orexin-A) Level Fluctuates with Season and Correlates with Day Length
Source: PLoS One. 2016 Mar 23;11(3):e0151288. doi: 10.1371/journal.pone.0151288 (PMC4805193; doi:10.1371/journal.pone.0151288)
Supplement: S5 Table — Summary of Multiple Regression Analysis. (DOCX) [file pone.0151288.s007.docx]

**Table S5**

No predictive value of C-reactive protein level. Summary of Multiple Regression Analysis.

| Variable | B | SE_B_ | β | *p*-value |
| --- | --- | --- | --- | --- |
| Intercept | 485.363 | 69.572 |  |  |
| Age | .333 | .290 | .103 | .254 |
| Gender | 4.118 | 9.163 | .041 | .654 |
| BMI | -2.158 | .961 | -.203 | .027 |
| Day length /3 weeks | .292 | .068 | .427 | .000042 |
| Snow | 56.584 | 16.173 | .333 | .001 |
| Days after Christmas | -5.279 | 2.316 | -.212 | .025 |
| CRP level | .812 | 1.220 | .061 | .507 |

B = unstandardized regression coefficient; SE_B_ = Standard error of the coefficient; β = standardized coefficient. N=109. *F*(7,101) = 4.805, *p* = 0.0001, R^2^ = 0.250.
